# Social Support and Symptoms of Depression in Late Life: Bidirectional Associations over Time

**DOI:** 10.3390/ijerph192316065

**Published:** 2022-11-30

**Authors:** Shira T. Turner, Sara Carmel, Norm O’Rourke, Victoria H. Raveis, Hava Tovel, Ella Cohn-Schwartz

**Affiliations:** 1 School of Public Health, Faculty of Health Sciences, Ben-Gurion University of the Negev, Be’er Sheva 84105, Israel; 2 Center for Multidisciplinary Research in Aging, Ben-Gurion University of the Negev, Be’er Sheva 84105, Israel; 3 Department of Psychology, Ben-Gurion University of the Negev, Be’er Sheva 84105, Israel; 4 Psychosocial Research Unit on Health, Aging and the Community, College of Dentistry, New York University, New York, NY 10010-2314, USA

**Keywords:** depression, late life, social support, structural equation modeling

## Abstract

Social support functions as an effective buffer against depression, especially among older adults with limited social networks. For the current study, we examined longitudinal bidirectional associations between social support and depression among those 75+ years of age. We recruited and followed a sample of Israeli adults 75+ years of age (N = 824; M = 80.84; range 75–96 years). Structured interviews were conducted in the homes of participants at three annual points of measurement. Participants reported depressive symptoms and emotional and instrumental support received from friends and family. We examined a cross-lagged, longitudinal structural equation model (SEM) in which social support and depressive symptoms predict each other over time, covarying for previously reported social support and depressive symptoms. We found that both depressive symptoms and social support are largely consistent in late life. Depressive symptoms and social support reported at baseline predict levels reported 1 and 2 years thereafter. Cross-over effects emerged over time. Depressive symptoms predicted lower social support in future, and social support at baseline predicted depressive symptoms 2 years later. These findings suggest that associations between depressive symptoms and social support are bidirectional in late life. Further research is needed to replicate findings in other cultures and over longer periods, ideally until end of life.

## 1. Introduction

Modern life has redefined living arrangements for most, as today more adults live alone and far from their families or origin [1]. The effects of these changes are especially pronounced for older adults with fewer social contacts [2,3]. This in turn affects both physical and mental health; in particular, depression, which is all too common in late life [4,5,6]. Previous research has shown that for older adults, social support serves as a protective factor [3] as the absence of social support increases the likelihood of depression [7]. Indeed, individuals with restricted social networks are at higher risk for depression [8].

Social support is defined as specific assistance or perceived general support [6,9] received from friends, partners and families [10]. Both instrumental or direct assistance and emotional support (e.g., love, trust) ameliorate depression [7,10]. Social support appears to directly affect both well-being and mental health [11] as those reporting more social support are less likely to feel lonely or depressed [5,6]. Bereaved older adults with strong social networks report less depression [2]. Additionally, healthy older adults who remain socially active are no more at risk for depression than young adults; that is, age alone is not a depression risk factor [5].

Though social support affects mental health, the opposite has also been reported. Schwartz and Litwin [12] found that the effects of both directions were significant yet the impact of mental health on social networks appeared significantly greater than the effects of social networks on mental health. A different longitudinal study found that among older adults undergoing treatment for depression, social support was high and increased over time [13]. Not only is overall social support important, but diversity of networks is also integral to mental health in late life [6]. More limited social networks are associated with higher levels of depressive symptoms.

Aside from social support, other factors are also associated with depression in later life. Older adults in good health [6,8] who are well educated [8,9] report less depression. Other research suggests no association between education and depression [2]. Poor health provides a pathway to depression [14] fostering loneliness and isolation, rather than vice versa [15].

Research conducted during the COVID-19 pandemic underscores the importance of social support in later life [16]. This period has been unprecedented in modern times, requiring young and old alike to shelter in place for extended periods [17]. Although many older adults were able to adapt and maintain social support, those with few meaningful connections were more likely to experience depression [16]. It also appears that perceived closeness but not social contact with relatives mediates the association between depression and loneliness; indeed, the risk of depression is inversely associated with both social network size [2] and perceived social support [16].

Social support has repeatedly been shown to predict depression in later life, yet most of the early research was conducted with young-old adults (i.e., 65–74 years). Today, many adults 75+ years of age actively work and maintain multiple social contacts. With advancing years, however, losses mount and social networks are reduced [6]. Moreover, most research has been cross-sectional, with data collected at one time only [18]. Additionally, most longitudinal studies have tested for one-way temporal associations, not bidirectional associations over time [5,19,20]. Longitudinal associations between social support and depression may differ in both size and direction over time.

For this study, we examined associations between social support and depression among those 75+ years of age. We hypothesized bidirectional associations between social support and depressive symptoms over time. Social support was assumed to be inversely associated with depressive symptoms at each point of measurement. Cross-lagged effects were assumed over time.

## 2. Materials and Methods

### 2.1. Participants and Procedures

We randomly identified adults 75+ years of age from Israeli Government records who lived in Haifa, Tel Aviv and Be’er Sheva. Prospective participants were contacted by phone and asked to respond to a short questionnaire. We selected those independent in activities of daily living, and able to respond in Hebrew or Russian (a high percent of older immigrants from the former Soviet Union remain more proficient in Russian than Hebrew). Interviews were conducted at three annual points of data collection: recruitment and 1 and 2 years thereafter.

A total of 1216 older adults were interviewed at baseline (T1; 32.7% in Haifa, 32.1% in Tel-Aviv, and 35.2% in Be’er Sheva), 1019 in the second wave of interviews (T2; 83.8% of the original sample), and 892 in the third wave (73.4%). Of those lost to attrition, 113 declined further participation, 131 became unable to participate (i.e., physical or cognitive disability), 60 died, and 20 could not be located after repeated attempts. After excluding a further 68 with missing socio-demographic data, 824 participants provided responses at each of the three points of annual data collection (67.8% of original sample).

At baseline, participants were 81.1 years old on average (SD = 4.03; range 75–96). Women comprised 44.6% of the sample and most (51.9%) lived alone, in contrast to comparatively few (19.7%) men. All participants were interviewed in their homes by research assistants trained to be sensitive to fatigue and concentration. Interviews were conducted in one or two sessions, lasting 1.5 to 3 hours.

### 2.2. Measures

The Berlin Social Support Scales (BSSS [21]) were administered to measure both perceived emotional support (4 items; e.g., “Every time I feel bad different people show me that they like me”) and perceived practical support (4 items; e.g., “Some people offer me help when I need it”). Responses were reported on a Likert scale ranging from *not true at all* (1) to *absolutely true* (5). Internal consistency was high at each point of data collection for both emotional (0.82. < α < 0.88) and instrumental support (0.86 < α < 0.91). See Table 1.

Depressive symptoms were measured using 6 items from the 15-item Geriatric Depression Scale (GDS-15 [22]) previously used in longitudinal research in Israel [23]. We began with a 4-item version of the GDS [24,25]. To increase internal consistency, two additional items from the original GDS were added (i.e., “Do you often feel helpless?” “Do you feel pretty worthless the way you are now?”). Strong Pearson correlation coefficients between the original GDS-15 and this 6-item version suggest little loss of validity (0.91 < r < 0.94 [23]).

Socio-demographic information collected included age, gender, religiosity, and household composition [6,8]. Education was measured as primary, secondary, post-secondary and academic [8,9]. Self-rated health was assessed by a single question measured using a Likert scale from *excellent* (0) to *very bad* (5).

### 2.3. Analytical Strategy

We hypothesized a longitudinal, structural equation model (SEM) in which social support and depressive symptoms predict each other concomitantly and in future (i.e., fully cross-lagged SEM). We covaried for prior measurement of both latent variables so that variance explained across latent constructs is unique. Error was assumed to be correlated across the same item pairs over points of measurement. Various sociodemographic variables associated with social support and depressive symptoms were first examined, and included if significantly associated with either latent construct. Descriptive statistics were computed with SPSS v28; SEM was computed with AMOS v26.

Consistent with convention, we report three goodness-of-fit-indices: an incremental, an absolute, and a parsimonious fit index. The Comparative Fit Index (CFI) is an incremental index representing the extent to which a hypothesized model is a better fit to data than the null model. The Standardized Root Mean Square Residual (SRMR) is an absolute index which represents the standardized difference between observed and predicted correlations within a hypothesized model. Finally, the Root Mean Square Error of Approximation (RMSEA) is a parsimony index which represents the extent to which a hypothesized model fits data relative to the population. Coefficient values greater than 0.94 for the CFI, and less than 0.055 for the SRMR and RMSEA, indicate good model fit [26].

## 3. Results

At baseline (T1), education was inversely associated with both social support and depressive symptoms. Perceived health also emerged as significantly associated with both constructs at baseline; both were added as covariates to the model. In contrast, neither sex, religiosity, living arrangement nor age emerged as significantly associated with either latent construct at recruitment. The latter may be due to the restricted age range of participants at recruitment (i.e., 75–96 years of age).

### Structural Equation Modeling

**Figure 1:**
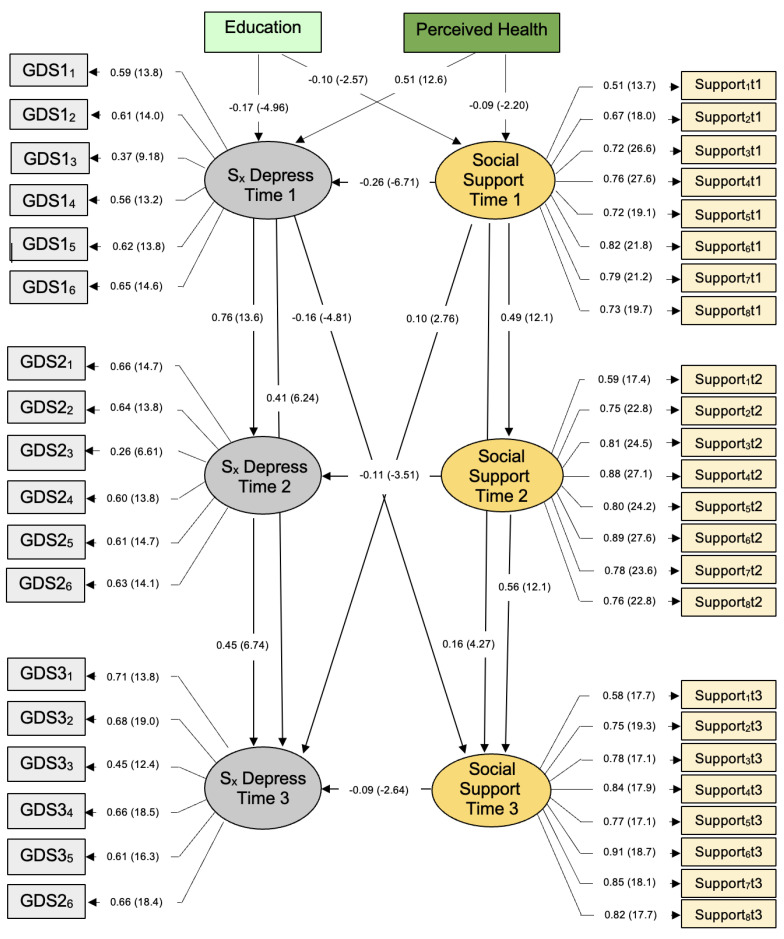
Social support and depressive symptoms over 2 years, Israelis 75+ years of age (N = 824). Parameters expressed as maximum likelihood estimates (standardized solution). Parenthetical numbers indicate significance levels, CR > |1.96|, *p* < 0.05; CR > |2.58|, *p* < 0.01.

The SEM that emerged differed from the fully cross-lagged model we hypothesized. After correcting for correlated error between four item pairs (i.e., different items), goodness-of-fit for this model was ideal, χ^2^(df = 850) = 1916.10, *p* < 0.01. More specifically, CFI = 0.95, SRMR = 0.043, and RMSEA = 0.039; the full 90% confidence interval for the RMSEA statistic was also in ideal parameters, 0.037 < RMSEA CL_90_ < 0.041. With 824 participants and 850 degrees of freedom, this model has sufficient statistical power (*d* = 0.99) to identify small effects (where α = 0.05 [26]). All statistically significant associations are shown in Figure 1; no other significant paths are not shown.

Each GDS item contributed significantly to measurement of depressive symptoms at each point of data collection (i.e., CR > |1.96|). The same also applied to responses to social support items, T1–T3. Social support at baseline predicted social support one year (T2) and two years thereafter (T3); the same emerged for depressive symptoms. These results suggest that both latent constructs are consistent in late life. Social support and depressive symptoms at baseline predicted responses one and two years thereafter.

Moreover, we observed a significant cross-over effect between depressive symptoms and social support. That is, depressive symptoms at baseline predicted lower social support 2 years later; the same was also true for social support at baseline (T1) which predicts depressive symptoms at T3. Cross-over effects were observed for neither latent construct after 12 months, not T1–T2. This cross-over effect between depressive symptoms and social support, and social support and depressive symptoms, emerged only 24 months later, or T1–T3.

Our findings corroborate existing research indicating that lower social support concomitantly predicts depressive symptoms in late life. Yet, cross-over effects between latent constructs suggest associations are more complex when examined over time. Social support and depressive symptoms appear to be interrelated phenomena in late life. Though largely consistent when measured over two years, cross-over effects between social support and depressive symptoms appear bidirectional when examined both concomitantly and over time.

## 4. Discussion

The results of this study corroborate previous research demonstrating links between social support and depression in late life. At each point of measurement, lower social support predicted higher depressive symptoms, adjusting for both previously reported social support and depressive symptoms (and education and perceived health at baseline). Longitudinal cross-over effects were also observed between social support and depressive symptoms, and depressive symptoms and social support, 2 years later. That is, depressive symptoms at baseline predicted lower social support not 1 year, but 2 years later.

Adjusting for both depressive symptoms and social support reported 1 and 2 years earlier, social support at baseline predicted depressive symptoms 2 years thereafter. The meaning of this result is not immediately apparent. If we revisit the model, however, note that social support at baseline predicted depressive symptoms at baseline and social support 1 and 2 years thereafter, as well as depressive symptoms 2 years thereafter. That is, remaining variance in social support that does not predict future social support represents lost social support at baseline that predicts depressive symptoms 2 years thereafter. This result is theoretically consistent in context.

Depressive symptoms and social support appear largely consistent in late life; levels of both at baseline predict levels reported 1 and 2 years thereafter. Over and above this consistency, contemporaneous effects of social support on depressive symptoms emerged at each point of measurement. Additionally, longitudinal cross-over effects were observed over 2 years. This pattern of findings suggests associations between depressive symptoms and social support are bidirectional over time. In other words, extended data collection is required in order to fully understand the associations between depression and social support; cross-sectional analyses may provide incomplete understanding of associations over time. Longitudinal measurement is integral, even with later life samples, despite attrition and other challenges.

The results of this study underscore the degree to which depression and social support are interconnected, at least in late life. Depression is defined on the basis of individual symptoms experienced within social networks of limited breadth and resilience. Our results indicate that social support is inversely associated with depressive symptoms at each point of measurement; moreover, lost social support (not reported in future) appears to predict depressive symptoms 2 years thereafter.

The implications of these findings for treatment and prevention underscore and reassert the role of instrumental and emotional support in late life. Support that is lost (i.e., not reported in future) predicts depressive symptoms today and in future. Similarly, reduced depression (i.e., not reported in future) predicts future but not contemporaneous social support. That is, the positive effects of reduced depression on social support in late life did not emerge contemporaneously, but only in future (i.e., 2 years later).

## 5. Limitations and Future Research

This study is unique in that we recruited and followed a large sample of older adults 75+ years of age. This allowed us to identify both contemporaneous and longitudinal associations between depressive symptoms and social support in late life. Participants lived in all regions of Israel (north, south and center); yet ethnic and religious minorities, and those living in small cities, were not included. Additionally, the limited age range of participants may have obscured the effects of age on depressive symptoms and social support in late life.

These results need to be replicated with other samples and other nations. Additionally, depressive symptoms and social support should be examined over an extended period, as our findings suggest that cross-over effects are observed only at 2 but not 1 year after initial measurement. Moreover, the direction of contemporaneous effects differed from longitudinal associations.

## 6. Summary and Conclusions

Both depressive symptoms and social support appear largely consistent in late life. Depressive symptoms and social support reported at baseline predict levels reported 1 and 2 years thereafter. Cross-over effects emerged over time. Depressive symptoms predict lower social support in future, and lost social support at baseline (not reported in future) predicts depressive symptoms 2 years later. These findings suggest that associations between depressive symptoms and social support are bidirectional in late life.

## Figures and Tables

**Table 1:** Study variables and descriptive statistics at recruitment, 1 and 2 years thereafter (N = 824).

Variables		Mean	SD	Skewness	Kurtosis	α
Berlin Social Support Scales	T1	34.67	6.41	−1.27	0.99	0.90
	T2	35.14	6.48	−1.53	2.11	0.93
	T3	35.16	6.49	−1.54	2.13	0.94
Geriatric Depression Scale	T1	4.96	1.46	−1.56	1.72	0.73
	T2	4.89	1.49	−1.43	1.28	0.72
	T3	4.83	1.65	−1.48	1.24	0.79

## Data Availability

Anonymized data are available from the corresponding author upon request.
